# Portable Amperometric Biosensor Enhanced with Enzyme-Ternary Nanocomposites for Prostate Cancer Biomarker Detection

**DOI:** 10.3390/bios14120623

**Published:** 2024-12-18

**Authors:** Thenmozhi Rajarathinam, Sivaguru Jayaraman, Chang-Seok Kim, Jaewon Lee, Seung-Cheol Chang

**Affiliations:** 1 Engineering Research Center for Color-Modulated Extra-Sensory Perception Technology, Pusan National University, Busan 46241, Republic of Korea; thenmozhi@pusan.ac.kr (T.R.); ckim@pusan.ac.kr (C.-S.K.); 2 Department of Cogno-Mechatronics Engineering, College of Nanoscience and Nanotechnology, Pusan National University, Busan 46241, Republic of Korea; sivaguru@pusan.ac.kr; 3 Department of Pharmacy, College of Pharmacy, Pusan National University, Busan 46241, Republic of Korea; neuron@pusan.ac.kr

**Keywords:** amperometric biosensor, sarcosine, prostate cancer, chitosan-polyaniline, mesoporous carbon, human serum, urine

## Abstract

Enzyme-based portable amperometric biosensors are precise and low-cost medical devices used for rapid cancer biomarker screening. Sarcosine (Sar) is an ideal biomarker for prostate cancer (PCa). Because human serum and urine contain complex interfering substances that can directly oxidize at the electrode surface, rapid Sar screening biosensors are relatively challenging and have rarely been reported. Therefore, highly sensitive and selective amperometric biosensors that enable real-time measurements within <1.0 min are needed. To achieve this, a chitosan–polyaniline polymer nanocomposite (CS–PANI NC), a carrier for dispersing mesoporous carbon (MC), was synthesized and modified on a screen-printed carbon electrode (SPCE) to detect hydrogen peroxide (H_2_O_2_). The sarcosine oxidase (SOx) enzyme-immobilized CS–PANI–MC-2 ternary NCs were referred to as supramolecular architectures (SMAs). The excellent electron transfer ability of the SMA-modified SPCE (SMA/SPCE) sensor enabled highly sensitive H_2_O_2_ detection for immediate trace Sar biomarker detection. Therefore, the system included an SMA/SPCE coupled to a portable potentiostat linked to a smartphone for data acquisition. The high catalytic activity, porous architecture, and sufficient biocompatibility of CS–PANI–MC ternary NCs enabled bioactivity retention and immobilized SOx stability. The fabricated biosensor exhibited a detection limit of 0.077 μM and sensitivity of 8.09 μA mM^−1^ cm^−2^ toward Sar, demonstrating great potential for use in rapid PCa screening.

## 1. Introductions

Prostate cancer (PCa) is a malignancy of the prostate gland. The Global Cancer Statistics 2022 (GLOBOCAN) report highlights PCa as a commonly detected cancer in 118 of 185 countries. In terms of cancer-related mortality among males, PCa ranks second in 52 countries. With an assessed 1.5 million new cases and 397,000 deaths in 2022, PCa was identified as the second most common cancer among males worldwide [1]. Ongoing efforts toward early PCa screening utilize molecular markers, prostate-specific antigen screening, standard biopsy, magnetic resonance imaging-targeted biopsy, digital rectal examination, and transrectal ultrasound. These clinical techniques are invasive, carried out by trained professionals, and yet expensive. In comparison with these clinical techniques, portable electrochemical systems coupled with disposable biosensors can offer countless benefits to potential users, including portability, low cost, quick readout, and miniaturization. The integration of these biosensors for “cancer-on-a-chip” platforms is an added advantage for their use in diverse sample matrices to meet current point-of-care needs [2]. Therefore, biosensors have become ubiquitous in the non-invasive point-of-care detection of target biomarkers. All healthy males have low levels of prostate-specific antigen (PSA), and elevated levels are linked to PCa, prostatic hypertrophy, and prostatitis. The PSA assay’s insufficient specificity and sensitivity are the cause of the high false-negative and false-positive readings [3]. Therefore, PSA alone is not an adequate biomarker, and there is an urgent need for alternative biomarkers to improve the PCa diagnostic accuracy. Sarcosine (Sar), which is elevated in the prostate tissue, blood, serum, and urine, is an important biomarker for PCa diagnosis. Normal Sar levels in the urine vary from 20.0 nM to 5.0 µM [4,5,6]. However, the determination of Sar is challenging because of the presence of potentially interfering substances in the serum or urine.

As a standard analytical method, liquid chromatography with tandem mass spectrometry (MS) [7] is not suitable for routine detection because of drawbacks such as the requirement for expensive instruments, lack of miniaturization possibilities, operational difficulties, complex sample pretreatment, and low sensitivity. Therefore, several challenges impede its transition to clinical practice. These challenges can be addressed by using simple, portable, fast, non-invasive, inexpensive, and reliable electrochemical biosensors [8,9]. Accordingly, the development of a portable Sar biosensor is a simple yet practically beneficial approach for fabricating point-of-care biosensors.

Mesoporous carbon (MC)-based nanomaterials are an important class of materials used to improve the electrocatalytic activity of biosensors. Their ability to promote fast electron transfer, stability, low ohmic resistance, sufficient biocompatibility, and improved interfacial adsorption properties make them unique for biosensor applications [10]. MC materials with distinct properties, morphologies, and rich N functionalities serve as peroxidase (POD)-like nanozymes to detect H_2_O_2_ [11,12]. Interfacing N-rich MC materials with suitable polymer matrices facilitates rapid H_2_O_2_ biomolecule detection [13]. In this context, chitosan (CS), an eco-friendly biopolymer, possesses high mechanical strength, hydrophilicity, and good film-forming ability. The chemical structural flexibility of CS and its amine linkages favor binding with enzyme biomolecules, thus expanding their applicability in enzymatic biosensors [14,15,16,17]. These unprecedented physiochemical properties of CS help enzyme biomolecules retain their original properties and become tightly entrapped on the electrode surface. Despite CS’s numerous physicochemical properties, its low electrical conductivity and instability limit its applications in biosensing [18]. Therefore, combining CS with other conductive polymers may enhance their electrocatalytic properties. Conductive polymers, such as polyaniline (PANI), polypyrrole, polyacetylene, and polythiophene, have been extensively investigated for biosensor fabrication. Among them, PANI has attracted considerable interest because of its facile synthesis, complex chemical structure, stability, and excellent electrical conductivity. CS exhibits limited conductivity, whereas PANI exhibits limited biocompatibility. To overcome these individual constraints, blending PANI with CS is a viable option as it is anticipated to improve the biocompatibility and electrocatalytic properties [19,20]. Therefore, CS–PANI hybrids are a burgeoning class of materials in which PANI imparts conductivity [21], while CS improves the biocompatibility and stability of enzyme biomolecules [22].

Khan et al. [18] synthesized CS–PANI nanohybrids to fabricate an immunosensor for ochratoxin A, thereby improving the surface morphology of PANI using CS. Covalent hydrogen bonding and extended π–π interactions between PANI and CS molecules increase the detection sensitivity. Yavuz et al. [23] developed a substituted CS–PANI sensor that could selectively detect glucose in the presence of active interferents. Yadav et al. [24,25] fabricated creatinine biosensors using the sarcosine oxidase enzyme (SOx) with Fe_3_O_4_/CS–PANI and ZnO/CS/MWCNT/PANI composites. Pandiselvi et al. [26] designed a sensor based on a multi-component CS–ZnO/PANI composite for the simultaneous determination of ascorbic acid (AA) and dopamine (DA) [27]. Thus, considering the significance of CS–PANI nanohybrids, biosensors integrated with these materials have great potential for the development of stable and reliable amperometric sensors.

**Scheme 1:**
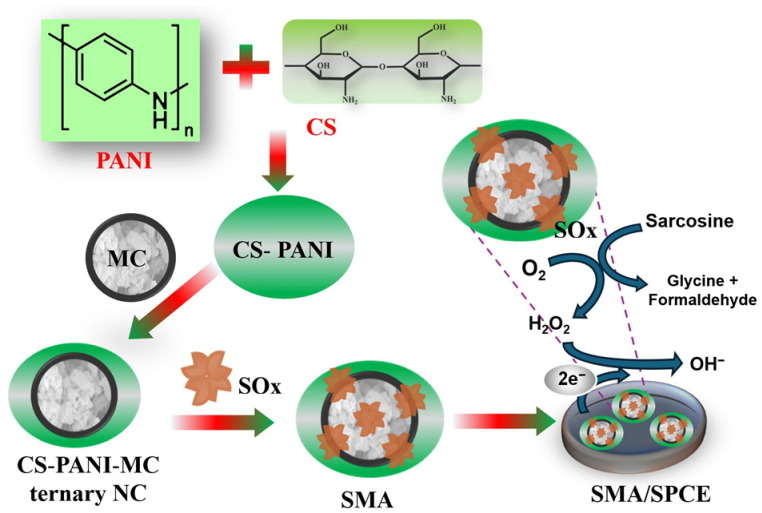
Schematic of the synthesized ternary nanocomposite, SMA/SPCE biosensor fabrication, and electrochemical Sar reaction. CS—stability, dispersibility, and glue-like properties for layer formation; PANI—high conductivity due to N functionalities; CS–PANI nanocomposite—combined properties of both CS and PANI; N-rich MC—H_2_O_2_ reaction byproduct sensing, high surface area for enzyme grafting; SOx—selective catalyst for Sar; SMA/SPCE biosensor—Sar concentration detection.

This study illustrates the development and utility of a metal- and reagent-free portable amperometric biosensor for Sar detection in human serum and urine samples. Initially, N-rich MC materials were synthesized via copolymerization, followed by template etching for efficient H_2_O_2_ sensing. SOx were immobilized on the CS–PANI–MC ternary NCs to form supramolecular architectures (SMAs) on a disposable screen-printed carbon electrode (SPCE) to fabricate an SMA/SPCE for selective Sar detection. The N-rich MCs stabilized by the CS–PANI hybrids imparted remarkable conductivity and improved specific surface area to entrap high SOx concentrations. The practical utility of the Sar biosensor was tested using human serum and synthetic urine samples to demonstrate its accuracy for Sar detection. In this study, the applied detection reaction (−0.2 V) was the reduction reaction of the produced H_2_O_2_ with the formation of hydroxyl ions. The SOx (Equation (1)) decomposed Sar to glycine, formaldehyde, and H_2_O_2_, and the produced H_2_O_2_ was reduced to hydroxyl ions (Equation (2) and Scheme 1):

(1) $$\mathrm{Sarcosine}+O_{2}+H_{2}O\;\overset{SOx}{\to }\;\alpha -\mathrm{Glycine}+\mathrm{Formaldehyde}\;{+H}_{2}O_{2}$$

(2) $$H_{2}O_{2}+2e^{-}\;\to {OH}^{-}$$

At −0.2 V, measuring the H_2_O_2_ produced in Equation (1) helped to indirectly estimate Sar concentrations.

## 2. Materials and Methods

### 2.1. Materials

Sarcosine oxidase (SOx, EC: 1.5.3.1) from bacillus species, Sar, sodium phosphate monobasic (NaH_2_PO_4_), sodium phosphate dibasic (Na_2_HPO_4_), potassium ferri/ferrocyanide [Fe(CN)_6_]^3−/4−^, H_2_O_2_, glucose (Glu), uric acid (UA), urea, ascorbic acid (AA), aniline (99%), toluene-4-sulfonic acid (PTSA), potassium chloride (KCl), ammonium persulfate (APS) (98%), acetic acid (99%), chitosan medium molecular weight (CS, 50.0–190 KDa, degree of deacetylation: 85%), ethanol (99.5%) sucrose, melamine, calcium carbonate (CaCO_3_), hydrochloric acid (HCl), and sodium chloride (NaCl) were all supplied by Sigma-Aldrich (Republic of Korea). Phosphate-buffered solutions (1.0 M) with different pH values were prepared using NaH_2_PO_4_, Na_2_HPO_4_, and deionized water (d.H_2_O) from a Milli-Q water purifier.

### 2.2. Instruments and Measurements

The details are provided in Section S.1 in the Supplementary Materials.

### 2.3. Synthesis of CS–PANI/MC Ternary NCs

#### 2.3.1. PANI Synthesis

PANI was prepared as described by Huang et al. [27]. 30.0 mL of 0.2 M PTSA was used to dissolve 0.56 g of aniline under continuous stirring and then cooled for 1 h. Then, APS (0.17 g dissolved in 0.2 M PTSA) was introduced slowly and continuously agitated for 3 h with the temperature being maintained at 5.0 °C with ice. The precipitated raw polymer was filtered and rinsed with d.H_2_O until it turned colorless. The final PANI product was dried at 50 °C for 12 h in an oven.

#### 2.3.2. Synthesis of the CS–PANI Polymer NCs

The CS/PANI polymer NCs were synthesized following Janaki et al. [28]. In this process, 0.25 g of CS was added to 25.0 mL of acetic acid (2.0% *v*/*v*) and agitated for 24 h at room temperature (25 °C). Approximately 15.0 mL of 0.10 M aniline (in 0.2 M PTSA) was added to this mixture and agitated for 15 min to obtain a homogeneous solution. Upon adding 5.0 mL of APS (0.15 M) solution, the polymerization process with CS was initiated under continuous stirring at 5.0 °C. The resulting mixture was agitated continuously for 6 h. After filtration, the resulting greenish-black product was washed with d.H_2_O until the filtrate became clear. The CS/PANI NCs were then dried in an oven at 50 °C for 12 h.

#### 2.3.3. Synthesis of the MC Materials

MC materials were synthesized using a copolymerization process followed by a template method, as reported by Peng et al. [29]. Briefly, sucrose (2.5 g) was added to d.H_2_O (20.0 mL), and later melamine and 5.0 g of CaCO_3_ were added under stirring, heated up to 80 °C for 2 h, and dried at 90 °C for 12 h. After reaching 25 °C, the product was washed with dilute HCl to remove any templates. The black products were collected via filtration, washed five times with d.H_2_O, and dried at 100 °C in a vacuum for 12 h. Thus, the MC materials (MC-1 and MC-2) were synthesized via the simultaneous copolymerization of sucrose and melamine in the presence of CaCO_3_ at 1200 °C. The samples were named MC-1 and MC-2, depending on the added amounts of melamine, i.e., 1.0 and 2.0 g, respectively.

#### 2.3.4. Synthesis of the CS–PANI–MC Materials

The protocol for the ternary composite synthesis was as follows. Approximately 0.50 g of CS was dissolved in 50.0 mL of acetic acid (2% *v*/*v*) and agitated for 24 h at 25 °C. Separate homogeneous dispersions of 50 mg MC-1 and MC-2 were prepared in 25.0 mL of 0.2 M PTSA under ultrasonication for 2 h. Both the MC and CS solutions were mixed under continuous stirring. Then, 25.0 mL of 0.1 M aniline was dissolved in 0.2 M PTSA and agitated for 15 min to form homogenous dispersions. APS solution (0.15 M) was also added to the reactants under constant stirring at for another 6 h. The obtained greenish-black product was washed, filtered, and dried at 50 °C for 24 h to acquire CS–PANI–MC-1 and CS–PANI–MC-2.

#### 2.3.5. Sar Biosensor Fabrication

The SPCEs were drop-coated with the synthesized CS–PANI–MC-2 ternary NCs pre-incubated with SOx, named SOx self-assemblies or SMAs. Because biosensor fabrication involves the simple physical adsorption of SOx onto ternary NCs, SMAs were formed. Therefore, the SMA/SPCE surface formed a stable and selective Sar sensing layer. The as-prepared biosensors were dried at 4.0 °C for 12 h and labeled SMA–SOx/SPCE.

#### 2.3.6. Real Sample Analysis

Human serum and synthetic urine samples were evaluated by the standard addition method to determine the efficiency of the biosensor in measuring total Sar concentrations. Human serum (H4522; male AB plasma) was acquired from Sigma-Aldrich and analyzed. Synthetic urine samples were prepared according to a previously reported method [30] as follows: calcium chloride (1.103 g), sodium chloride (2.295 g), sodium sulfate (2.25 g), potassium phosphate (1.40 g), potassium chloride (1.60 g), ammonium chloride (1.00 g), urea (25.0 g), and creatinine (1.10 g). The concentrations in Sar-spiked serum and urine samples were also determined following the chronoamperometry (CA) conditions described in the Experimental Techniques Section.

## 3. Results and Discussion

### 3.1. Physicochemical Characterizations of the NCs

**Figure 1:**
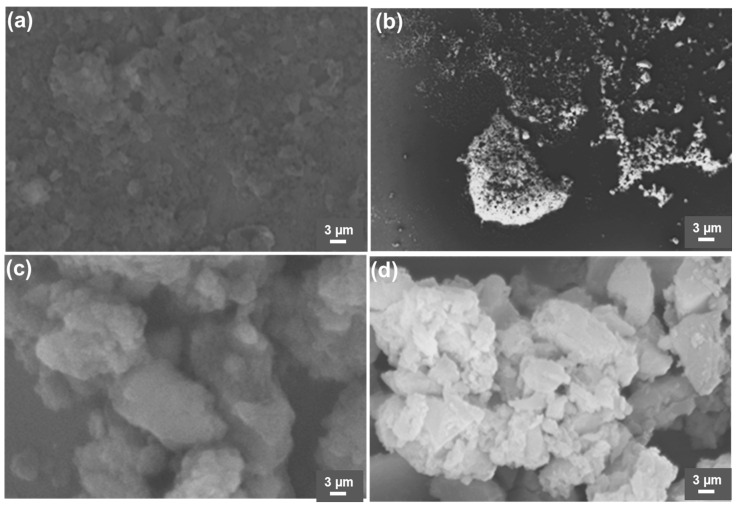
FE–SEM images of the NCs (**a**) PANI, (**b**) CS–PANI, (**c**) CS–PANI–MC-2, and (**d**) SMA. Scale: 3.0 µm.

The synthesized NCs were examined via field emission scanning electron microscopy (FE-SEM), and the results are shown in Figure 1. PANI and CS–PANI exhibited granular structures distributed on slightly smooth surfaces (Figure 1a,b) similar to the study [31]. The CS–PANI–MC-2 (Figure 1c) exhibited a distinct tiny spherical morphology of MCs covered with irregular polymeric granules of CS and PANI. After CS–PANI–MC-2–SOx (SMA) drop casting, the surface appeared very smooth, and the spherical morphology was seen to be disturbed (Figure 1d), suggesting the effective self-assembly of the SOx enzyme and CS–PANI–MC ternary NCs.

**Figure 2:**
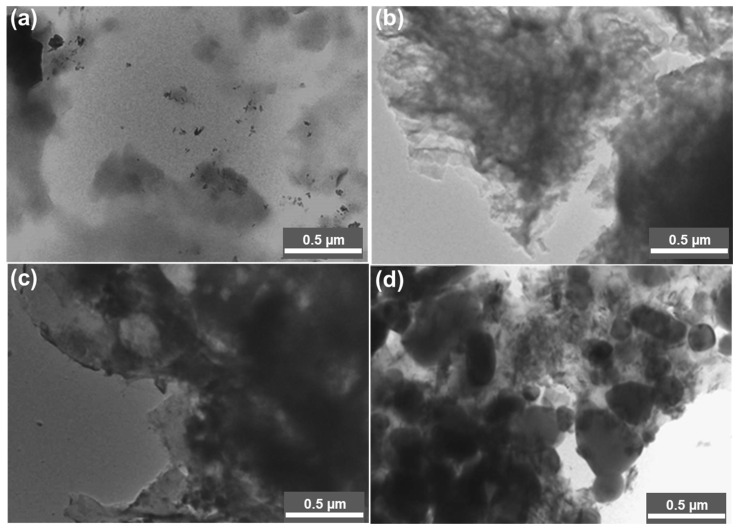
TEM images of the synthesized NCs: (**a**) PANI, (**b**) CS–PANI, (**c**) CS–PANI–MC-1, (**d**) CS–PANI–MC-2. Scale: 0.5 µm.

**Figure 3:**
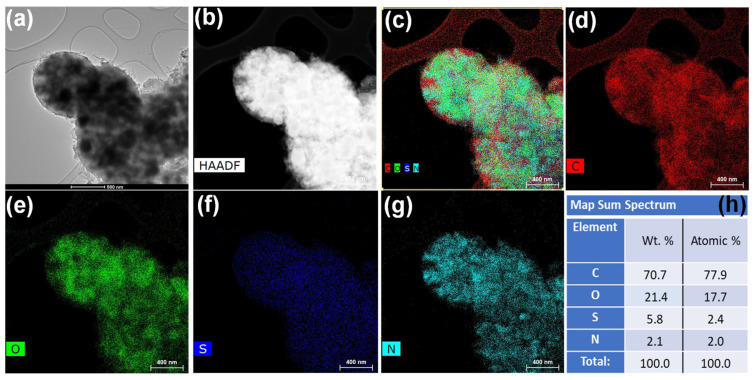
(**a**) HRTEM image of CS–PANI–MC-1 NCs at a 500 nm scale. (**b**,**c**) HAADF-STEM showing the existence of C, O, S, and N. (**d**–**g**) Individual elemental maps of C, O, S, and N. (**h**) Elemental wt. % and atomic %.

**Figure 4:**
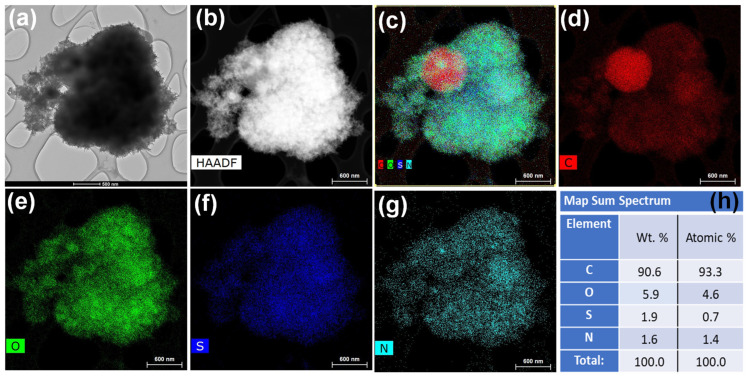
(**a**) HRTEM image of CS–PANI–MC-2 NCs at a 500 nm scale. (**b**,**c**) HAADF-STEM images. (**d**–**g**) Corresponding individual elemental maps. (**h**) Elemental wt. % and atomic %.

The transmission electron microscopy (TEM) images (Figure 2) of PANI and CS–PANI indicated the presence of irregular granules, a typical characteristic of both CS and PANI polymers, whereas the TEM images of CS–PANI–MC-1 and CS–PANI–MC-2 revealed the typical spherical morphology of MCs [32] covered with irregular CS–PANI polymeric granules. Additionally, high-resolution TEM images of CS–PANI–MC-1 (Figure 3) and CS–PANI–MC-2 (Figure 4) revealed the presence of spherical MCs covered with CS–PANI layers. High-angle annular dark-field scanning TEM (HAADF–STEM) coupled with energy-dispersive X-ray (EDX) spectra showed that C, O, S, and N were evenly distributed over the MC spheres. The atomic percentages of C, O, S, and N in CS–PANI–MC-1 were 77.9%, 17.7%, 2.4%, and 2.0%, respectively (Figure 3). The S functionalities were due to H_2_SO_4_ involvement during the MC synthesis, and N functionalities of 2.0% were due to the N-doping of MC materials and CS–PANI polymers. The low number of N functionalities implied that the remaining N could be strongly encapsulated inside the MC matrix. The EDX spectrum of PANI–MC-2 (Figure 4) also illustrated the presence of similar C, O, S, and N elements with atomic percentages of 93.3%, 4.6%, 0.7%, and 1.4%, respectively. The CS–PANI polymers rendered the MC materials more hydrophilic, thereby enabling the formation of self-assembled layers. The hydrophilicity and dispersion stability of CS–PANI–MC-2 were attributed to its N and S functionalities [33].

Figure S1 presents the Fourier transform infrared spectroscopy (FTIR) spectra of PANI, CS–PANI, CS–PANI–MC-1, and CS–PANI–MC-2. For PANI (red line), the characteristic FTIR bands observed at 1556 cm^−1^ and 1462 cm^−1^ corresponded to the N=Q=N stretching vibrations of the quinoid ring (Q) and the NH–B–NH stretching of the benzenoid ring (B). The intensity ratio of the Q/B bands was approximately one, confirming the presence of doped PANI. Additionally, the band at 1290 cm^−1^ was attributed to the C–N stretching of the aromatic amine group, while a shoulder at 1200 cm^−1^ was associated with C–N^+^ bonds of the polaronic framework. In the FTIR spectra of CS–PANI NCs (blue line), a series of peaks were observed, shifted relative to the PANI bands, indicating strong interactions between PANI and CS on the composite formation. Additional peaks between 442.5 and 922.1 cm^−1^ corresponded to C–H stretching, while the peak at 1044.0 cm^−1^ arose from the C–O stretching vibrations of glycosidic linkages in CS. The spectra of CS–PANI–MC-1 (green line) and CS–PANI–MC-2 (violet line) NCs displayed all the characteristic peaks of CS–PANI NCs, along with an additional C=C stretching peak at 1636 cm^−1^. This feature confirmed the incorporation of porous carbon networks into the composites [31,32,33].

### 3.2. Electrochemical Tests of the Biosensors

**Figure 5:**
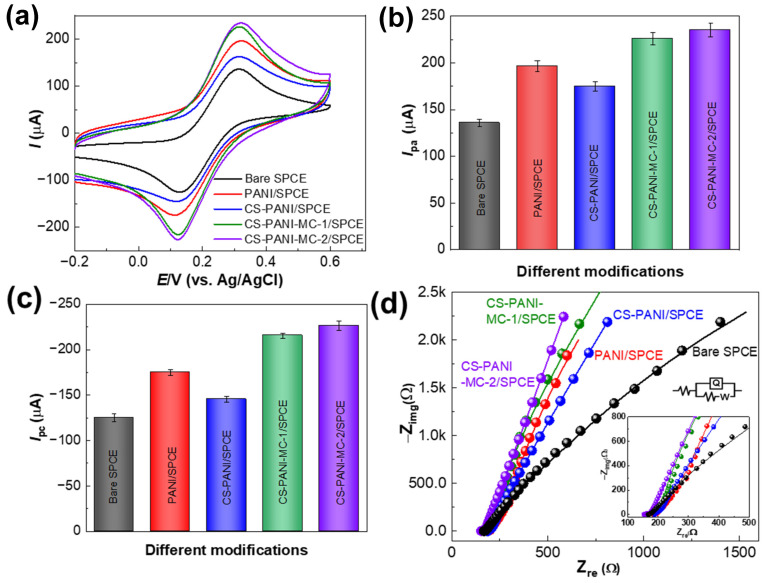
(**a**) CVs of the modified biosensors in a 5.0 mM [Fe(CN)_6_]^3−/4−^ probe at a 50 mV s^−1^ scan rate. (**b**,**c**) *I*_pa_ and *I*_pc_ plots. (**d**) EIS results. Inset: the zoomed-out portion of the EIS and the fitted Randles circuit, R(Q(RW)).

The electrochemical properties of Bare SPCE, PANI/SPCE, CS–PANI/SPCE, CS–PANI–MC-1/SPCE, and CS–PANI–MC-2/SPCE were examined via cyclic voltammetry (CV) analysis using an [Fe(CN)_6_]^3−/4−^ redox couple. As shown in Figure 5a, all biosensors exhibited inherent redox peaks. The bar graph in Figure 5b shows the lowest anodic peak current (*I*_pa_) (135.7 µA) for Bare SPCE, indicating a slow electron transfer at its surface, which might be due to the presence of organic moieties [34]. Electron transport was fast after the PANI modification onto the SPCE, which exhibited an *I*_pa_ value of 196.4 µA. The CS–PANI/SPCE displayed a slightly decreased *I*_pa_ value of 163.3 µA compared with the PANI/SPCE due to the slight insulating properties of CS. However, the presence of CS was essential because it could form a stable sensing layer. A further improvement in *I*_pa_ values was evident for CS–PANI–MC-1/SPCE (225.8) and CS–PANI–MC-2/SPCE (235.1), owing to the abundant delocalized π-electrons in the sp^2^ carbon network of MC and π-electron clouds of CS–PANI. The sufficient synergy between the CS–PANI polymers and MC is shown in Figure 5c; the cathodic peak current (*I*_pc_) values of Bare SPCE, PANI/SPCE, CS–PANI/SPCE, CS–PANI–MC-1/SPCE, and CS–PANI–MC-2/SPCE were −125.2, −174.9, −145.4, −215.3, and −226.4 µA, respectively. As shown in Figure 5b,c, the *I*_pa_ and *I*_pc_ values of CS–PANI–MC-2/SPCE were the highest, indicating the fast electron transfer properties of the material, attributed to the synergistic impact of the highly conductive MC and PANI. The ratio of *I*_pc_ and *I*_pa_ was nearer to −1, and the redox peak separation was much larger than 57 mV, indicating that the electrochemical reaction at the electrode interface was a quasi-reversible type [35].

Electrochemical impedance spectroscopy (EIS) was used to study the electrical properties of the biosensors. The Nyquist impedance plots of the biosensors are shown in Figure 5d. The EIS results fit well with the Randles circuit *R*(*Q*(*RW*)). The first component of the circuit was the electrolyte solution resistance (*R_s_*) [34]. The small semicircle in the high-frequency region indicated that the biosensor resistance was due to the charge transfer resistance (*R_ct_*), and the vertical line was specific to the Warburg impedance (Z_w_) [36,37] due to the transport of ions that diffused through the porous architecture of the MC. The *R_ct_* values of Bare SPCE, PANI/SPCE, CS–PANI/SPCE, CS–PANI–MC-1/SPCE, and CS–PANI–MC-2/SPCE were 325, 284, 292, 276, and 265 Ω, respectively. The *R_ct_* values decreased regularly after PANI and MC modifications, and the CS–PANI–MC-2/SPCE showed the smallest *R_ct_* (265 Ω), indicating considerable improvement in the charge transfer ability of the CS–PANI–MC-2 materials. Compared with Bare SPCE, the Z_w_ value of CS–PANI–MC-2/SPCE increased by 70%, making it a suitable matrix for the electrochemical sensing of the target. Additionally, the constant phase element (*C_CPE_*) values of the biosensors were 1630, 1896, 1890, 1900, and 1928 µF cm^−2^. The changes in the Z_w_ and *C_CPE_* values were influenced by the CS–PANI and CS–PANI–MC ternary NCs layers. The enhanced capacitance and decreased *R_ct_* values indicated the highly conductive nature of the CS–PANI and porous MC networks.

The surface electrocatalytic activities and charge transfer characteristics were quantitatively evaluated by determining the active surface area (ASA) using Randles–Ševčík’s equation [9,34]. As depicted in Figure S2, the peak currents (*I*_p_) exhibited a linearity with the square root of the scan rate (ν^1/2^), signifying a diffusion-controlled electron transfer process at the sensor. Notably, as the scan rate increased from 10 to 300 mV s^−1^, well-defined redox peaks were observed (Figure S2a), with a corresponding increase in *I*_p_. The linear correlation between *I*_p_ and ν^1/2^, as shown in Figure S2b,d,f,h, further supported the diffusion-controlled kinetics at the biosensor interface. The calculated ASA values for Bare SPCE, PANI/SPCE, CS–PANI/SPCE, CS–PANI–MC-1/SPCE, and CS–PANI–MC-2/SPCE were 0.122, 0.126, 0.130, 0.135, and 0.138 cm^2^, respectively. These results demonstrated enhanced electrocatalytic activity, particularly for CS–PANI and CS–PANI–MC composites. The improvements in *I*_pa_, *I*_pc_, and ECSA values confirmed the superior conductivity of the CS–PANI matrix and the porous carbon networks in CS–PANI–MC-2/SPCE. Additionally, the roughness factor ratio (ASA/geometrical area) of 1.1 highlighted the presence of catalytically active sites in CS–PANI–MC-2.

### 3.3. Chronoamperometric H_2_O_2_ Detection

**Figure 6:**
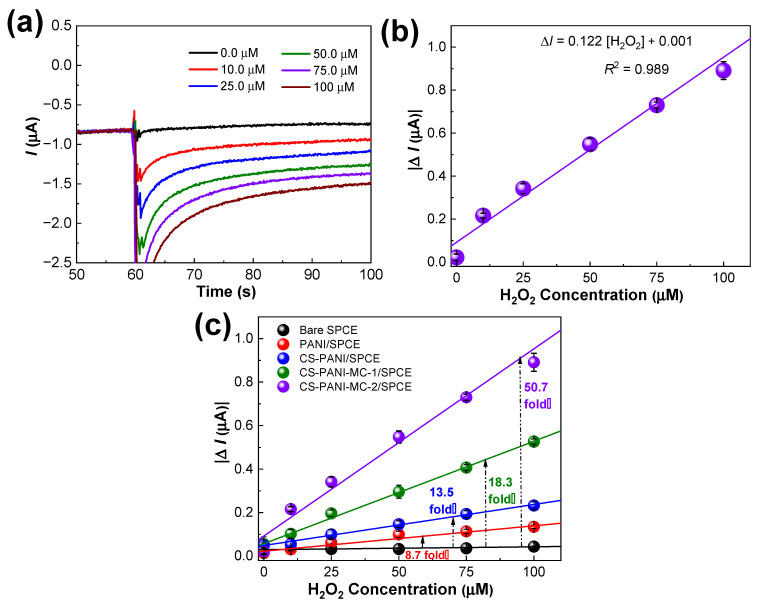
(**a**) CA responses of CS–PANI–MC-2/SPCE against different H_2_O_2_ concentrations (0, 10, 25, 50, 75, and 100 µM) in PBS pH 7.5. (**b**) Calibration and (**c**) linear plots acquired for the modified biosensors via CA with successive H_2_O_2_ additions (SD ± 4).

Chronoamperometry (CA) was tested for Bare SPCE, PANI/SPCE, CS–PANI/SPCE, CS–PANI–MC-1/SPCE, and CS–PANI–MC-2/SPCE against different H_2_O_2_ concentrations in a buffer solution, and the current responses measured after 30 s are displayed as calibration plots in Figure 6. The Bare SPCE exhibited negligible responses, whereas the PANI/SPCE and CS–PANI/SPCE exhibited a slight increase in current with increasing H_2_O_2_ concentration. The CS–PANI–MC-2/SPCE exhibited the highest current response toward H_2_O_2_, as shown in Figure 6a, and its linear plot (Figure 6b) with the regression equation *I* = 0.122 [H_2_O_2_ (µM)] + 0.001 yielded a correlation coefficient of 0.989. The sensitivities of the Bare SPCE, PANI/SPCE, CS–PANI/SPCE, PANI–MC-1/SPCE, and CS–PANI–MC-2/SPCE were 0.95, 8.28, 12.82, 17.36, and 48.20 µA mM^−1^ cm^−2^, respectively. The limit of detection (LOD) for H_2_O_2_ was 1.20 µM. The sensitivity of CS–PANI–MC-2/SPCE was higher than that of the other sensors owing to the synergism between CS and PANI and MC-2. The improved H_2_O_2_ sensing of CS–PANI–MC-2/SPCE stemmed from the high surface area and conductivity of PANI–MC, which in turn was due to accelerated electron transfer [38]. As illustrated in Figure 6c, the sensitivities of the PANI/SPCE, CS–PANI/SPCE, PANI–MC-1/SPCE, and CS–PANI–MC-2/SPCE were 8.7, 13.5, 18.3, and 50.7 times higher than that of the Bare SPCE, respectively.

### 3.4. SMA/SPCE Optimization

The produced SMA/SPCE biosensor was optimized for SOx loading concentrations (0.1, 0.2, 0.4, 0.6, 0.8, and 1.0 mg/mL) with CS–PANI–MC-2 to acquire self-assembled monolayers or SMAs of CS–PANI–MC-2 and SOx. The CA responses were measured 30 s after 5.0 µM Sar additions. As shown in Figure S3a, the CA responses are enhanced by increasing the enzyme loadings from 0.1 to 0.8 mg/mL. By contrast, the CA response at the highest enzyme loading (1.0 mg/mL) decreased because of enzyme overloading in the CS–PANI–MC-2 matrix. As the highest CA response was attained for 0.8 mg/mL SOx, it was taken as the optimal concentration for SMA layer formation. The optimum solution pH was determined using a phosphate buffer (0.1 M) at a pH of 6.0–8.0. The maximum CA response to 5.0 µM Sar additions was obtained for pH = 7.5 and was chosen as the suitable working pH (Figure S3b). The potential was optimized via CA tests on the SMA/SPCE against 5.0 µM Sar at applied potentials from −0.20 to 0.0 V. Based on the results (Figure S3c), an applied potential of −0.20 demonstrated stronger current responses than the other applied potentials.

Thus, the CS–PANI–MC-2 materials exhibited enhanced stability and intrinsic catalytic performance toward H_2_O_2_. The presence of SOx in the SMA/SPCE biosensor resulted in superior catalytic activity and robust Sar-sensing stability. These findings provide a simple yet straightforward approach for the rational fabrication of a biomimetic SMA/SPCE, which could enable the development of feasible electrochemical biosensors for rapid diagnostic devices.

### 3.5. Sar Calibration Plots

**Figure 7:**
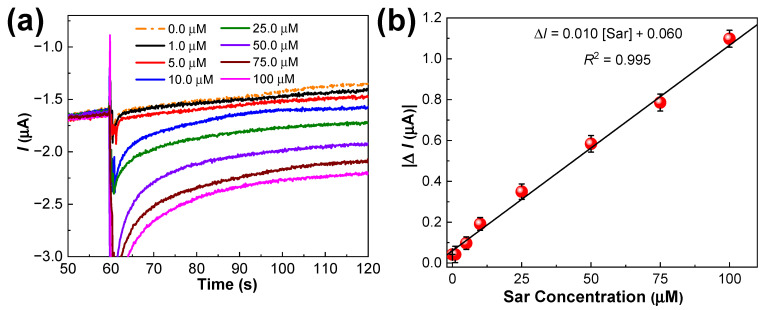
(**a**) CA responses of the SMA/SPCE at the −0.20 V potential for different Sar concentrations. (**b**) Linear plot showing the CA responses vs. Sar concentrations.

The response time was the time required for the biosensor to produce a CA response after Sar addition, which had a vital influence on its efficacy. To examine the response time, CA was performed by adding Sar to the buffer solution. There was a sharp increase in the current response after the addition of Sar using the SMA/SPCE under the optimized experimental conditions, and the resulting calibration curve was plotted using the amperometric responses after 30 s of Sar addition. As shown in Figure 7, the biosensor exhibited a linearity between 1.0 and 100 µM with an LOD of 0.077 µM (S/N = 3 at 95.0% confidence interval (CI)), the LOD being calculated following [36]. The regression line was as follows: *I* (µA) = 0.010 [Sar (µM)] + 0.060, with a correlation coefficient of 0.995. The LOD was low, surpassing some biosensors listed in Table 1 [38,39,40,41,42,43], and the linear range was wider than that of certain previously reported biosensors [40,41,42,43]. The biosensor had proven advantages in terms of detecting ultra-low levels of Sar and could be readily applied to complicated sample matrices such as urine.

### 3.6. Selectivity

**Figure 8:**
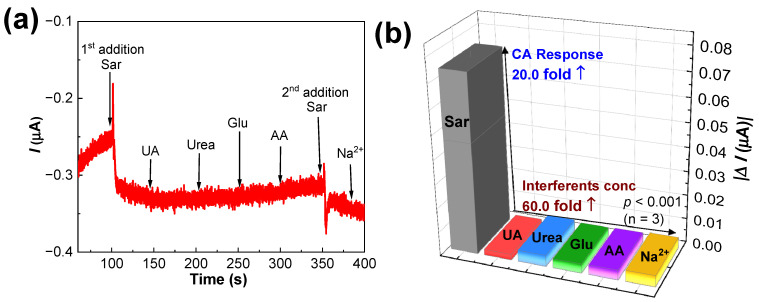
(**a**) CA results of the SMA/SPCE biosensor in optimized conditions upon 5.0 µM Sar additions, 60-fold (300 µM) higher concentrations of UA, urea, Glu, AA, and Na^2+^ ions. (**b**) CA values plotted after baseline subtraction.

The selectivity for Sar against co-interferents was tested, and the CA results are shown in Figure 8a. The SMA/SPCE biosensor elicited a quick CA response to Sar (5.0 µM), while the co-interferents, such as uric acid (UA), urea, glucose, AA, and sodium ions, exerted no interferences, even at 60-fold higher concentrations (300 µM). The second Sar additions (at 350 s) retained nearly 50% of the initial Sar signal. Particularly, the addition of the co-interferents produced weak CA responses, with less than 5.0% of the Sar signal, as shown in Figure 8b. The amine functionalities of the PANI–CS polymer and sulfur functionalities from the MC provided a negative charge at the biosensor interface, which excluded unwanted signals from electronegative UA and AA biomolecules. Glucose is a polar molecule that does not donate electrons at a pH of 7.5. Interference from undesirable enzymatic reactions is an issue in Sar biosensors because SOx is a highly specific catalyst [9,39,43]. The selectivity coefficient (*k*_sel_) was calculated as follows:

(*k*_sel_) = (Signal)_interferent_/(Signal)_Sar_ (3)

The *k*_sel_ value of the co-interferents was <0.001, which confirmed the high selectivity of the biosensor toward Sar (Table 2). The non-interaction of the SMA/SPCE (*n* = 3) with the aforementioned co-interferents was also validated using a paired *t*-test. The *t*-test results revealed that the SMA/SPCE was not influenced by co-interferents.

### 3.7. Stability and Reproducibility

**Figure 9:**
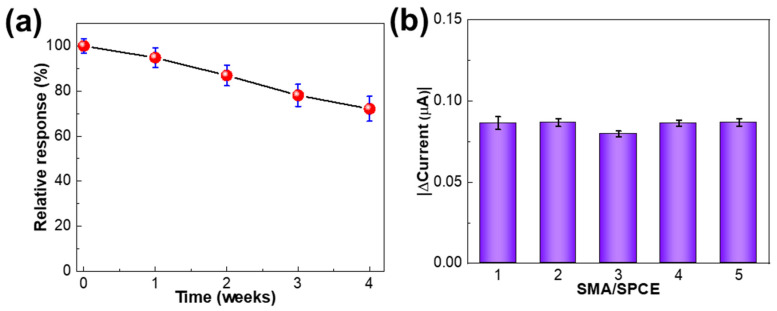
(**a**) Long-term storage stability of the SMA/SPCE at 4.0 °C over 4 weeks and (**b**) its reproducibility (*n* = 5). The error bars represent the mean of three measurements obtained using five biosensors.

Stability is an important measure of the sensor’s electrochemical performance. To examine the stability of the SMA/SPCE, the current responses toward 5.0 µM Sar were measured on the first, second, third, and fourth weeks, and the results are shown in Figure 9. The biosensors (*n* = 5) were kept at 4.0 °C and tested weekly against 5.0 µM Sar. As shown in Figure 9a, the losses in CA response after 1, 2, 3, and 4 weeks were 5.2%, 13.1%, 21.9%, and 27.9%, respectively (SD ± 5). This loss in CA response was due to the passivation or disappearance of some enzymes. Nevertheless, approximately 72.1% of the initial CA response to Sar was retained even after four weeks, corroborating the robust stability of the SMA/SPCE. The reproducibility of the biosensors (*n* = 5) was investigated via CA experiments, as shown in Figure 9b. The relative standard deviation (RSD) of five identically produced biosensors was 3.4%. In particular, sufficient intermolecular interactions between the ternary NCs and SOx biomolecules considerably enhanced the reproducibility of the biosensor.

### 3.8. Sar Testing in Real Samples

Human serum and synthetic urine samples were spiked with different Sar concentrations to evaluate the biosensor performance. The samples were prepared by spiking the samples with a known amount of Sar (1.0–20.0 µM). The results obtained at these four concentrations are listed in Table 3. The recovery ratios obtained for the serum and synthetic urine samples were >95.0% and 96.0%, respectively, corresponding to RSD errors of less than 5.0% and 3.5%.

## 4. Conclusions

In this study, a simple, disposable, and portable enzymatic biosensor based on the self-assembly of SOx on CS–PANI–MC-2 ternary NCs was developed for Sar detection in serum and synthetic urine. Electrostatic interactions and charge transfer at the redox center of SOx enabled efficient Sar detection using the SMA/SPCE. Therefore, the enzymatic redox reaction was favorable owing to the extended stability and biocompatibility of the ternary NCs. The proposed biosensor exhibited a linearity of 1.0–100 μM in the clinically relevant Sar concentrations found in PCa patients, which could facilitate its application in cancer-on-a-chip assay platforms. Additionally, the exhibited high sensitivity of 8.06 μA mM^−1^ cm^−2^ and low LOD of 0.077 μM was attributable to the synergistic properties of CS–PANI and MC materials. Since the biosensor accurately measured Sar levels in serum and synthetic urine samples, the propounded portable biosensor is a suitable Sar screening tool in both laboratory and non-laboratory settings.

## Figures and Tables

**Table 1:** Comparison of the Sar biosensor parameters with other amperometric biosensors.

Biosensor	Applied Potential (V) vs. Ag/AgCl	Linear Range (µM)	LOD (µM)	Real Samples Tested	Reference
SMA/SPCE	−0.2 V	1.0–100	0.077	Human serum and synthetic urine	This work
SOxENs/PB/SPCE	−0.1 V	10.0–400	0.66	Human urine	[9]
SOx–PVA–Au–pphTEOS	0.55 V	500–7500	500	-	[38]
SOx–Pt–MNP/GCE	0.4 V (SCE)	5.0–40	0.24	Human serum	[39]
SOx–Nano Pt–ZIF8	0.4 V (SCE)	5.0–30	1.06	-	[40]
SOx–Pt–MTP/GCE	0.25 V (SCE)	1.0–71.0	0.40	Human serum	[41]
SOx–Fe–N–C/GCE	0.3 V (SCE)	2.0–37.0	0.70	Human serum	[42]
SOx/SPCE	0.6 V	0.01–0.10	0.016	Synthetic urine	[43]
SOx–MX–CS/GCE	−0.7 V	0.036–7.80	0.018	Synthetic urine	[44]
SOx–PAA/GCE	−0.55 V	0.001–0.05	0.0004	Human urine	[45]
SOx/SPCE	0.6 V	0.01–0.10	0.016	Synthetic urine	[46]
SOx–CS–CuNPs–c–MWCNT/Au	0.2 V	0.10–100	0.00001	Human serum	[47]
SOx–Pt–OIHMMP/GCE	0.3 V (SCE)	1.0–70	0.13	Human serum	[48]
SOx–PANI	0.4 V (SCE)	100–1000	-	-	[49]
SOx–PPy	0.4 V (SCE)	100–1000	-	-	[50]

SOxENs, SOx enzyme nanosheets; PB, Prussian blue; SOxENPs, SOx NPs; MX, carbides and nitrides of transition metals; GCE, glassy carbon electrode; PAA, polyamic acid; PVA, polyvinyl alcohol; pphTEOS, partial prehydrolyzed tetraethyl orthosilicate; c-MWCNT, carboxylated multi-walled carbon nanotubes; SCE, standard calomel electrode; OIHMMP, organic–inorganic hybrid molybdenum phosphonate; Fe_3_O_4_@C, iron (II,III) oxide@carbon; Pt–NEAs, platinum-nano electrode arrays; PPy, polypyrole; MTP, mesoporous tungsten phosphonate; Fe–N–C, iron-nitrogen-doped carbon; MNP, mesoporous nickel phosphonate; ZIF8, zeolitic imidazolate framework-8.

**Table 2:** Effect of interferents studied using *k*_sel_ values and *t*-test results.

Target + Interferent	Current (µA)	Interferent	Current (µA)	*k*_sel_	Paired *t*-Test Interference
Sar	0.07394	-	-	-	-
Sar + UA	0.07631	UA	0.00237	0.0320	No
Sar + UA + Urea	0.08102	Urea	0.00471	0.0637	No
Sar + UA + Urea + Glu	0.08491	Glu	0.00389	0.0526	No
Sar + UA + Urea + Glu + AA	0.08903	AA	0.00412	0.0557	No
GLU + DA + NE + Glucose + AA + Na^2+^ ions	0.09374	Na^2+^ ions	0.00471	0.0637	No

**Table 3:** Sar concentrations analyzed in spiked human serum and artificial urine samples (*n* = 4).

Samples	Added (µM)	Found (µM)	Recovery (%)	RSD (%)
Human serum	-	-	-	-
	1.0	0.98 ± 0.01	98.0	4.5
	2.5	2.43 ± 0.05	97.1	1.3
	5.0	4.84 ± 0.01	96.8	0.7
Synthetic urine	10.0	9.53 ± 0.03	95.3	3.3
	-	-	-	-
	1.0	0.99 ± 0.04	99.5	3.4
	2.5	2.46 ± 0.04	98.5	2.5
	5.0	4.86 ± 0.03	97.2	3.4
	10.0	9.65 ± 0.01	96.5	3.3

RSD: relative standard deviation.

## Data Availability

Data are contained within the article and Supplementary Materials.

## Supplementary Materials

The following supporting information can be downloaded at https://www.mdpi.com/article/10.3390/bios14120623/s1: Section S1: Instrumentation and Measurements. Figure S1: FTIR spectra of the materials; Figure S2: (a,c,e,g) Cyclic voltammograms of Bare SPCE, PANI/SPCE, CS–PANI/SPCE, and CS–PANI–MC-2/SPCE in 5.0 mM [Fe(CN)_6_]^3−/4^ at 10–300 mVs^−1^ scan rates and (b,d,f,h) respective *I*_pa_ and *I*_pc_ calibration plots; Figure S3: (a) SOx optimization with ternary NCs, (b) buffer pH optimization, and (c) applied potential optimization.
