# Supplementary material for: Portable Amperometric Biosensor Enhanced with Enzyme-Ternary Nanocomposites for Prostate Cancer Biomarker Detection
Source: Biosensors (Basel). 2024 Dec 18;14(12):623. doi: 10.3390/bios14120623 (PMC11675002; doi:10.3390/bios14120623)
Supplement: Supplementary file 1 [file biosensors-14-00623-s001.zip › biosensors-3309110-supplementary.pdf]

Supporting Information

Portable Amperometric Biosensor Enhanced with Enzyme-Ternary Nanocomposites for Prostate Cancer Biomarker Detection

Thenmozhi Rajarathinam[1,2†], Sivaguru Jayaraman[2†], Chang-Seok Kim[1,2], Jaewon Lee[3], and Seung-Cheol Chang[2*]

[a] Engineering Research Center for Color-Modulated Extra-Sensory Perception Technology, Pusan National University, Busan 46241, Republic of Korea; thenmozhi@pusan.ac.kr (T.R.)
2 Department of Cogno-Mechatronics Engineering, College of Nanoscience and Nanotechnology, Pusan National University, Busan 46241, Republic of Korea; sivaguru@pusan.ac.kr (S.J.) ckim@pusan.ac.kr (C.-S. Kim)
3 Department of Pharmacy, College of Pharmacy, Pusan National University, Busan, 46241, Republic of Korea; neuron@pusan.ac.kr (J.L.).

†These authors contributed equally to this work.

* Corresponding author.

Department of Cogno-Mechatronics Engineering, College of Nanoscience and Nanotechnology, Pusan National University, Busan 46241, Republic of Korea

Email:

s.c.chang@pusan.ac.kr (S-C. Chang).

S.1. Experimental Techniques

Surface characterization of the materials was performed using field-emission scanning electron microscopy (FE-SEM, Zeiss SUPRA 25) with energy-dispersive X-ray spectroscopy (EDS). High-resolution transmission electron microscopy (HRTEM) images were acquired using a HITACHI H–7600 200 kV instrument (Hitachi High–Tech Corp., Tokyo, Japan). Fourier-transform infrared (FT-IR) spectroscopy was conducted using a Thermo Scientific Nicolet iS50 spectrometer (Thermo Fisher Scientific Korea Ltd., Seoul, South Korea) after forming pellets with potassium bromide (KBr). Cyclic voltammetry (CV) and electrochemical impedance spectroscopy (EIS) were conducted using a potentiostat (CH Instruments, USA, model 604E). Chronoamperometry (CA) measurements were carried out using a portable potentiometer (Sensit Smart, PalmSens Technologies B.V, Houten, Netherlands) with a connecting software, PSTrace (PalmSens BV, Houten, Netherlands) for data acquisition and analysis. An inexpensive smartphone connected *via* the Bluetooth mode was used to observe and record the CA signals.

SPCE (Model name: C11L) procured from Metrohm DropSens (Oveido, Spain) was utilized as a base electrode material. The geometric area of the working electrode in the SPCE was 0.1256 cm^2. Approximately 6.0 μL of SMA were drop-cast over the working electrode surface of the SPCE. SPCE consisted of an internal Ag/AgCl as the reference electrode, and a carbon auxiliary electrode. A 2.0 mL disposable cell was set up with the biosensor for the CV experiments. The biosensor was immersed in 2.0 mL of 0.1 M KCl containing 5.0 mM $[Fe(CN)_6]^{3-/4-}$ redox probe with potential sweeps from −0.2 to +0.6 V at a 50 mV s^{-1} scan rate and different scan rates from 10 to 300 mV s^{-1}. For EIS analysis, in the $[Fe(CN)_6]^{3-/4-}$ probe, the biosensor was kept at a frequency range of 100 kHz to 0.10 Hz, which is the AC potential amplitude (5.0 mV) on a DC potential (250 mV). For CA, the biosensor was connected *via* a portable potentiostat, approximately 60.0 μL of PBS was placed on the biosensor surface and

polarized at −0.20 V. After reaching a stable baseline response, 20.0 μL of the Sar solution was added at 60 s, and the current responses were measured after 30 s. The calibration curves were plotted using CA measurements (n = 4) with various concentrations of Sar.

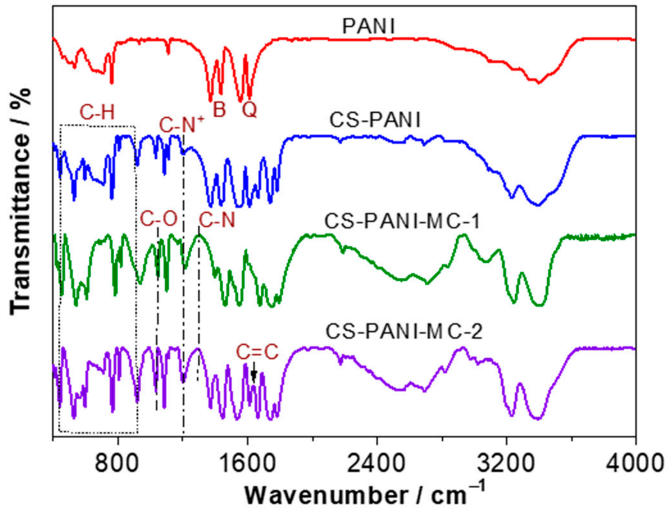

Figure S1. FT-IR of the synthesized NCs, PANI, CS–PANI, CS–PANI–MC–1 and CS–PANI–MC–2.

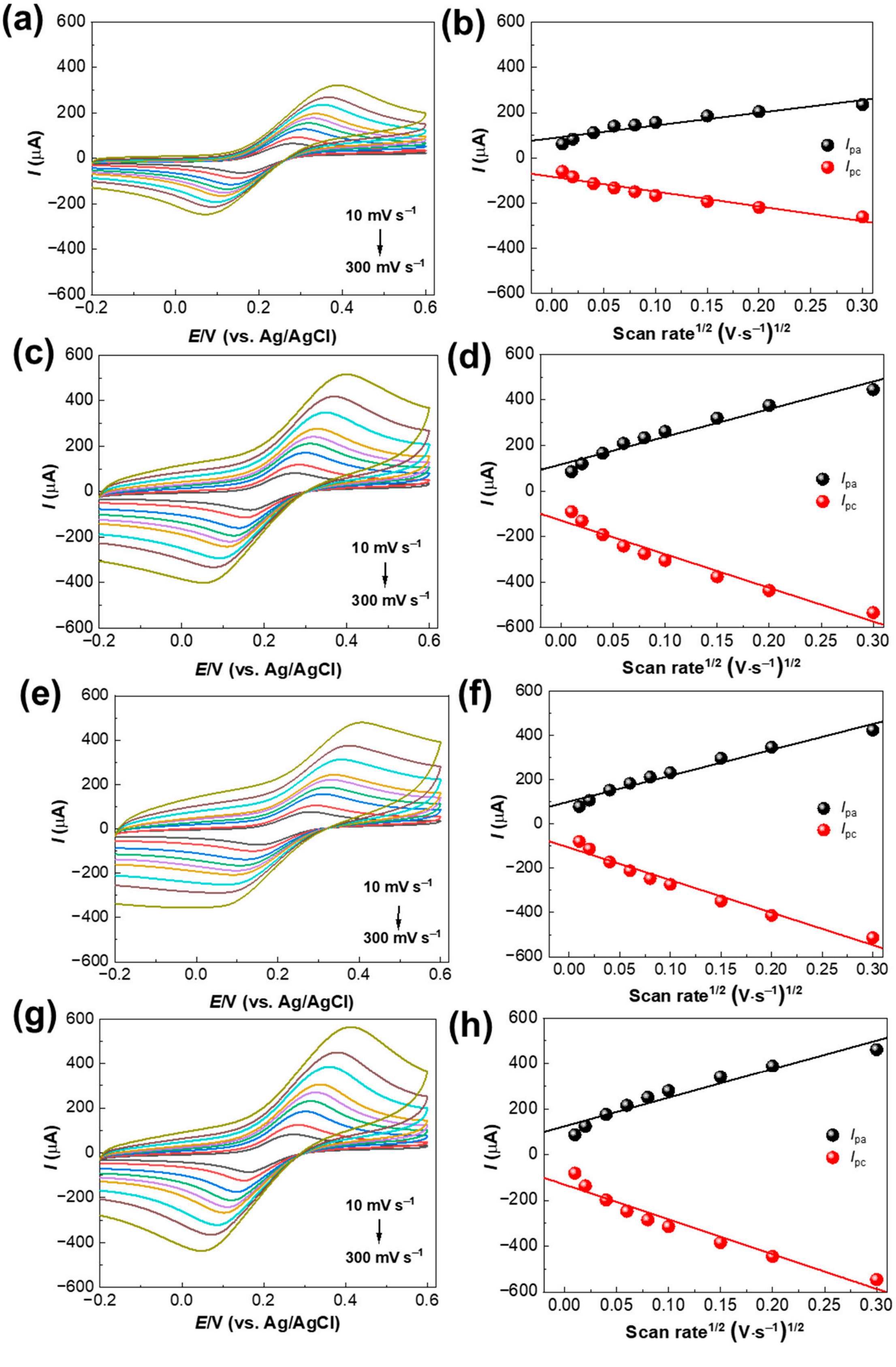

Figure S2. (a, c, e, & g) CVs of Bare SPCE, PANI/SPCE, CS–PANI/SPCE, and CS–PANI–MC–2/SPCE in 5.0 mM $[Fe(CN)_6]^{3-/4-}$ in 0.1 M KCl at increasing scan rates (10 to 300 mVs^{-1}) and (b, d, f, & h) respective I_{pa} , I_{pc} calibration plots.

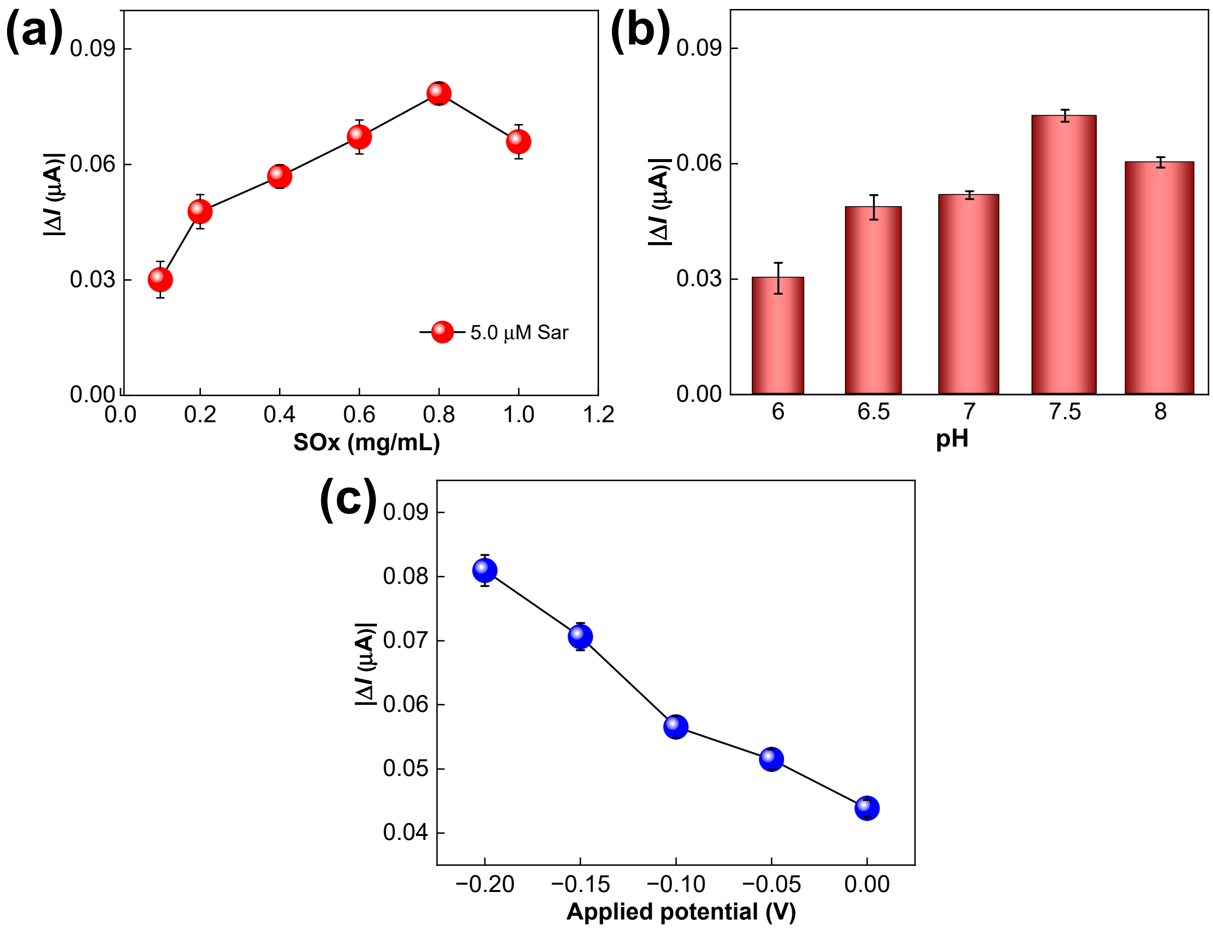

Figure S3. Optimization of SOx enzyme, solution pH, and applied potential. (a) Current responses for 5.0 μM Sar on SMA/SPCE fabricated using various concentrations of SOx enzyme (0.1, 0.2, 0.4, 0.6, 0.8, and 1.0 mg SOx. (b) Current changes obtained for varied pH solutions (pH 6.0, 6.5, 7.0, 7.5, and 8.0) on SMA/SPCE in 5.0 μM Sar. (c) Current changes measured for various applied potentials (–0.2, –0.15, –0.1, –0.05, and 0.0 V) on SMA/SPCE.
